# The role of Card9 overexpression in peripheral blood mononuclear cells from patients with aseptic acute pancreatitis

**DOI:** 10.1111/jcmm.12738

**Published:** 2015-12-21

**Authors:** Zhi‐wen Yang, Cheng‐zhao Weng, Jing Wang, Ping Xu

**Affiliations:** ^1^ Department of PharmacySongjiang Hospital Affiliated Shanghai First People's HospitalShanghai Jiao Tong UniversityShanghaiChina; ^2^ Department of DigestiveShanghai Songjiang Hospital Affiliated to Nanjing Medical UniversityNanjingChina; ^3^ Department of DigestiveSongjiang Hospital Affiliated Shanghai First People's HospitalShanghai Jiao Tong UniversityShanghaiChina

**Keywords:** Card9 overexpression, peripheral blood mononuclear cells, acute pancreatitis, non‐infection inflammation

## Abstract

Activated mononuclear cells are an early event in the course of severe acute pancreatitis (SAP). To date, the molecular mechanism triggering peripheral blood mononuclear cells (PBMCs) is poorly understood. The aim of this paper was to determine the potential role of Card9 in SAP. We collected data from 72 subjects between January 2013 and June 2014. Subsequently, PBMCs were isolated on day 1, 3 and 5 of pancreatitis. Immunofluorescence staining, quantitative real‐time PCR, Western blotting, immunoprecipitation and ELISA were used to determine the role of Card9 in SAP. Microbial culture showed that SAP patients at the early period did not develop any bacteria and fungi infection. Card9 expression in SAP patients was higher than that in mild acute pancreatitis and volunteer healthy controls, up to the peak on day 1. The monocyte‐derived cytokines interleukin (IL)‐17, IL‐1β, IL‐6 and tumour necrosis factor‐α mediated by the induction of Card9 markedly increased in SAP patients compared with the control group. Furthermore, the inducible formation of Card9‐Bcl10 complex was found in PBMCs, which may be involved in nuclear factor kappa B (NF‐κB) and p38 activation in SAP. Receiver operating characteristic curve indicated that Card9 levels had a high sensitivity of 87.5% and specificity of 67.7%, showing the close correlation with SAP patients. Card9 overexpression was firstly found in aseptic SAP, which may be played an important role in NF‐κB and p38 activation in PBMCs. It also provided the new insights into therapeutic interventions by targeting monocytes activation in SAP patients.

## Introduction

Severe acute pancreatitis (SAP) is a disease with a high mortality rate ranging from 10% to 30% due to rapid systemic organ failure 1. Apart from the supportive therapy, no effective active treatment exists in clinical practice. It is currently accepted that the activated mononuclear cells locally and systemically are an early event in the course of SAP, inducing inflammatory cascade reaction and developing systemic organ failure in patients 2, 3, 4. To date, the molecular mechanism leading to the initiation and development of the activated mononuclear cells still remains incompletely defined in SAP 5, 6.

Caspase recruitment domain 9 (Card9) is an adaptor protein that is mainly expressed in monocyte‐macrophage cells and assists in the integration of inflammation signalling pathways downstream of pattern recognition receptors 7, 8. Recent studies demonstrate that Card9 expression in mononuclear cells serves as vital immune inflammation responses for defence against bacterial and fungal infection 9. For example, Card9 deficiencies have been found to cause recalcitrant subcutaneous pheohyphomycosis infections in patients, with reduced TH17 cells and impaired immune responses against P verrucosa 10. However, the early course of AP is characterized by non‐infectious inflammation. Thereby, it is unknown whether Card9 adaptor molecule is required for the early course of acute pancreatitis without microbial infection.

We previously showed that the inhibition of NF‐κB and p38 mitogen‐activated protein kinase (MAPK) signalling molecule could attenuate the severity of SAP in human acute monocytic leukaemia (THP‐1) cell and rat model 11, 12. Given the role of Card9‐dependent NF‐κB and MAPK activation in infectious inflammation, we further have been suggested that Card9 overexpression might be an upstream signalling molecule of NF‐κB and p38 MAPK to activate the mononuclear cells in SAP.

In this study, Card9 in SAP patients was overexpression, suggesting the close correlation with the outcome and severity of pancreatic injury in patients. Furthermore, Card9 overexpression was involved in NF‐κB and p38 MAPK activation in peripheral blood mononuclear cells (PBMCs) from SAP patients. For the first time, our preliminary findings linked Card9 adaptor molecule with the susceptibility to non‐infectious inflammation, which enriched the diseases spectrum underlying Card9 signalling pathways other than the previously reported microbial infections.

## Materials and methods

### Patients and definitions

The study protocol was approved by the committee for the Ethical Issue in Songjiang Hospital Affiliated Shanghai First People's Hospital, Shanghai Jiao Tong University, and informed consent form was obtained from each patient. In this prospective study conducted at this hospital between January 2013 and June 2014, all patients with AP were collected to enter the study. We assessed 52 consecutive patients on day 1, 3 and 5 pancreatitis, including 35 mild acute pancreatitis (MAP) and 17 SAP within 24 hrs after the onset of abdominal pain symptoms. Patients with the sign of chronic pancreatitis or past history of AP were excluded. The control group consisted of 20 healthy volunteers.

The diagnosis of acute pancreatitis was confirmed according to the following features, *i.e*. typical abdominal upper epigastric pain, serum lipase levels >3 times the upper limit of normal standard and imageological examination 13. Clinical severity of acute pancreatitis was categorized retrospectively by use of Ranson's criteria, a traditional method that evaluated inflammation or organ failure 14.

Blood samples from each patient were collected to isolate PBMCs on day 1, 3 and 5 after the symptom onset, while samples from healthy volunteers were only taken once. Before the clinical therapy, blood were collected and regarded as the first day samples of their symptom onset. Simultaneously, microbal infection including bacteria and fungus was analysed using blood culture technology. Laboratory data of all patients were recorded in detail, such as bacterial culture, leucocyte and biochemical indexes.

### PBMCs isolation

Peripheral blood mononuclear cells were isolated by Ficoll‐Paque density gradient centrifugation (GE Healthcare, Fairfield, CT, USA) from blood samples of patients or volunteer controls. Briefly, 10 ml of peripheral blood was pooled into an ethylenediaminetetraacetic acid anti‐coagulant tube, after which tube was diluted with Ficoll‐Paque solution and centrifuged. Subsequently, PBMCs were retained at the interface between the plasma and Ficoll‐Paque, where they could be collected and added into a sterile tube. Considering the fact that monocytes exhibited the adherent characteristics, the isolated PBMCs were further incubated for 4 hrs at 37°C with 5% carbon dioxide to remove the non‐adherent lymphocytes. Finally, the purified PBMCs were carefully transferred to a new tube for the following study.

### Immunofluorescence staining

Peripheral blood mononuclear cells were labelled with Card9 antibodies according to the manufacturer's instructions (Thermo, Waltham, MA, USA). Peripheral blood mononuclear cells pooled from each patient were subsequently fixed and permeabilized by use of formaldehyde/xylene solution. After hydration through alcohol at different concentration, PBMCs were incubated overnight with rabbit polyclonal antibody to Card9 (1:500) at 4°C. Next, cells were incubated with FITC‐conjugated goat anti‐rabbit IgG antibody (1:500) for 40 min. at 37°C in the dark. Negative staining as a control group was conducted in parallel with the omission of primary antibodies.

### Quantitative Real‐time PCR

qRT‐PCR was carried out to assay Card9 and Bcl10 mRNA expression in PBMCs using reverse transcription system kit (Thermo) according to the manufacturer's instructions. In short, total RNA were extracted immediately from freshly isolated PBMCs using a Trizol reagent (Invitrogen, Carlsbad, CA, USA). Then, 1 μg of total RNA was reverse‐transcribed to the first‐strand cDNA using miRNA RT kit for each sample. The first‐strand cDNA was used as template for PCR amplification with qPCR Master Mix. Samples were amplified in the ABI Prism 7300 SDS Software (Applied Biosystems Foster City, CA, USA) using the SYBRs Green PCR Mix (Thermo) at the following conditions: 95°C for 10 min., 40 cycles of 95°C for 15 sec. and 60°C for 45 sec. The following primer pairs (Generay, Shanghai, China) were used: Card9‐F (GCAGGTGTTCCAGTGTGAG)/Card9‐R (GGCAGCCTTTGTCTGAGAG); Bcl10‐F (GGAGTGTGAGCCACCTAAG)/Bcl10‐R (CTGGGCGATAGAGCAAGAC); GAPDH‐F (AATCCCATCACCATCTTC)/GAPDH‐R(AGGCTGTTGTCATACTTC).

### Western blotting

Western blotting was used to monitor Card9 and Bcl10 protein expression in PBMCs. Cells were homogenized at a cell density of 1 × 10^7^ cells/50 μl. 20–40 μg of total protein was loaded on 10% polyacrylamide gel, and the proteins were transferred to a nitrocellulose membrane. After blocking, the membranes were incubated with rabbit polyclonal anti‐Card9 antibody, β‐actin, NF‐κB, p38, rabbit polyclonal anti‐Bcl10 antibody overnight at 4°C, and then with goat anti‐rabbit secondary antibody for 60 min. at 37°C. Bands were visualized with enhanced chemilumescent (ECL) estern blotting substrates, analysed by Gel imaging system (Bio‐Rad, Hercules, CA, USA). Finally, the grey value was calculated by the quantity one software.

### Immunoprecipitation

Peripheral blood mononuclear cells from individual patient were immunoprecipitated with 20 ml anti‐HA 3F10 beads according to the manufacturer's instructions. The cellular lysates were collected and added into agarose. The obtained immunoprecipitates and lysates were subjected to SDS‐PAGE followed by electroblotting onto a polyvinylidenedifluoride membrane (Millipore Boston, MA, USA) using the indicated antibodies.

### Inflammation factor

Serum samples were collected from patients and stored at −70°C until used for analyses. Inflammation factors were determined by using commercially available ELISA kits according to the manufacturer's instructions (JRDUN, Shanghai, China).

### Statistical analysis

To predict the diagnostic value of Card9 levels, receiver operating characteristic (ROC) curve analysis was plotted and calculated. Statistical analysis was performed using SPSS 20.00 software (SPSS Inc., Chicago, IL, USA). Quantitative variables were described using mean ± S.E.M., and the differences were analysed by *t*‐test. Nonparametric distribution data were expressed as median (interquartile range), and the differences were analysed by Mann–Whitney *U*‐test. Correlations analysis was carried out using Spearman's rank test. *P*‐value less than 0.05 was considered statistically significant.

## Results

### Patient data

Based on Ranson score, the severity of AP was classified as mild in 35 patients (67.3%), and severe in 17 patients (32.7%). Table 1 showed the aspartate transaminase, lactic dehydrogenase, C‐reactive protein, white blood cell, aetiology and clinical characteristics of patients with acute pancreatitis. Microbal infection including bacteria and fungus was analysed in AP patients using the first day samples of their symptom onset. A total of 52 patients did not show the bacterial and fungal infection in blood, indicating that patients who presented with non‐infectious inflammation were collected in this study. After receiving the hospital therapy for 5 days, most patients could greatly alleviate the symptom and gain a recovery. On day 5 of pancreatitis, bacterial infection could not happened or worsen but rather inflammation resolution, along with disease recovery. In this study, one SAP patient died and three SAP patients suffered from multiple organ failure. They still showed the negative microbial culture on day 3 because few SAP patients could develop microbial infection at the initial phase (days 0 to 3).

**Table 1:** Characteristics of AP patients

	MAP (*n* = 35)	SAP (*n* = 17)	Control
Age (year)	49.86 ± 2.65	68.29 ± 3.73	39 ± 3.60
Males:females (*n*)	17:18	12:5	9:11
Ranson score	1.16 ± 0.18	3.14 ± 0.18	–
Deaths	0	1	–
Multiple organ failure	0	3	–
Aetiology			
Gallstones	24	13	–
Alcoholism	0	0	–
Hyperlipidaemia	5	1	–
Idiopathic	6	3	–
AST (mmol/l)	106.0 ± 25.1	169.2 ± 51.5	26.95 ± 2.94
LDH (IU/l)	221.4 ± 22.1	365.7 ± 41.4	–
CRP (mg/l)^a^	54.0 (30.5, 106.5)^a^^,^ b	77.4 (17.4, 138.2)^b^	–
WBC (×10^9^/l)	13.4 ± 0.9	17.0 ± 1.6	7.17 ± 0.25
Bacterial culture	Negative	Negative	–
Fungal culture	Negative	Negative	–

a SAP *versus* MAP, *P* < 0.05.

b Nonparametric distribution, interquartile range method.

The first day samples of their symptom onset were used to analyse the serum biochemical index and microbial culture (*n* = 52). On day 3 and 5, clinical data were not listed.

AST, aspartate transaminase; LDH, lactic dehydrogenase; CRP, C‐reactive protein; WBC, white blood cell.

### Card9^+^ PBMCs were markedly induced in patients with SAP

**Figure 1:**
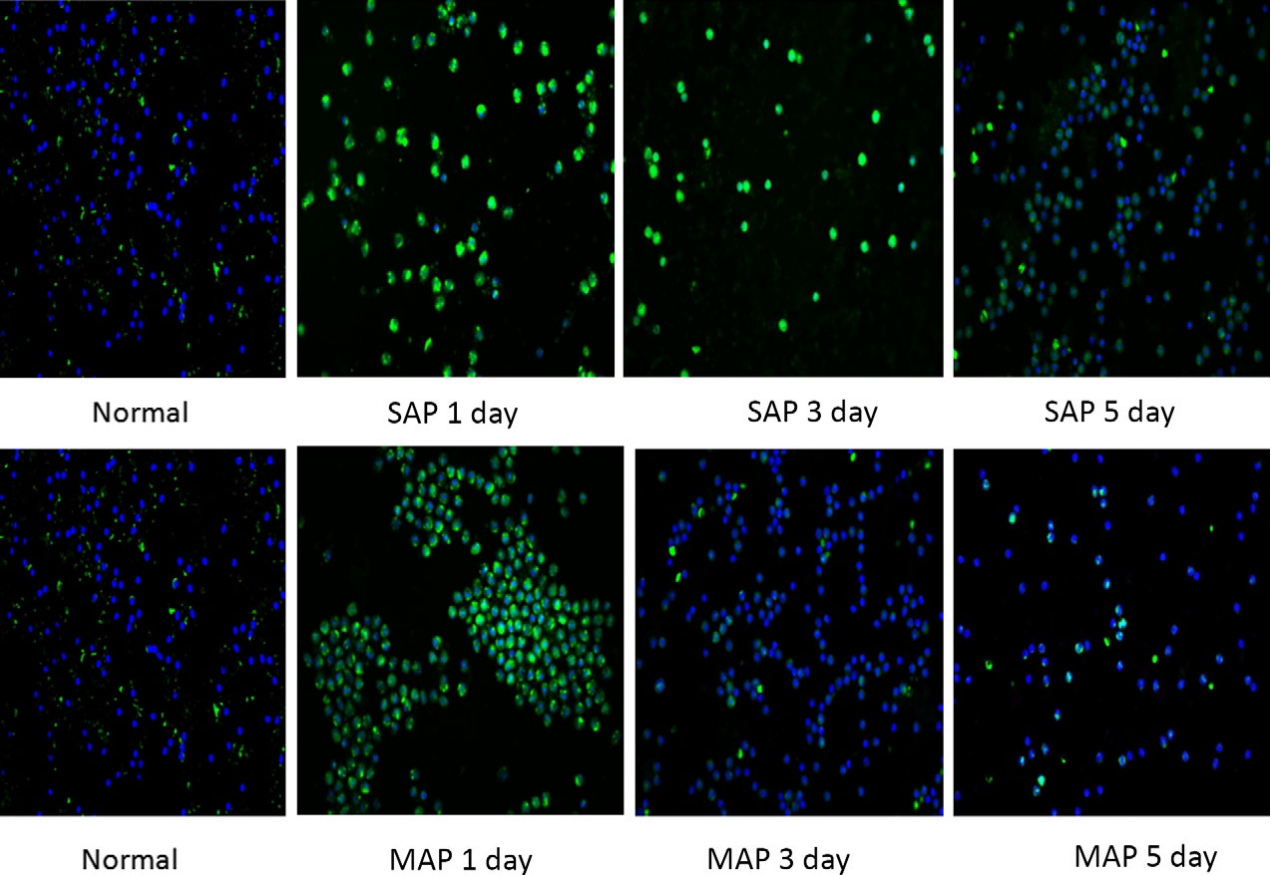
Immunofluorescence staining of Card9 expression in PBMCs. These results were representative from 20 healthy volunteers, 35 MAP and 17 SAP patients. Green fluorescence and blue fluorescence represented Card9 and cellular nucleus respectively.

To investigate Card9 levels in the development of SAP, immunofluorescence staining was used to measure Card9 expression in PBMCs. Card9 data from patients were described in Figure 1. On day 1, 3 and 5, Card9 fluorescence intensity in AP patients was significantly higher than healthy volunteers. Specifically, all PBMCs from SAP patients exhibited the strong green fluorescence, and green fluorescence was very full in each cell. However, about 80–90% of PBMCs from MAP patients exhibited the strong green fluorescence, and green fluorescence was not full in each stained cell. Thus, immunofluorescence staining clearly revealed that Card9 levels markedly increased in SAP compared with MAP, which was associated with disease severity. In addition, an obvious decrease in Card9 level on day 3 was found among MAP and SAP patients after they received the standardized treatment including fasting, gastrointestinal decompression, H_2_ receptor blocking agent, water‐electrolyte balance, homoeostasis, symptomatic treatment, prophylactic administration of antibiotics and alimentotherapy.

**Figure 2:**
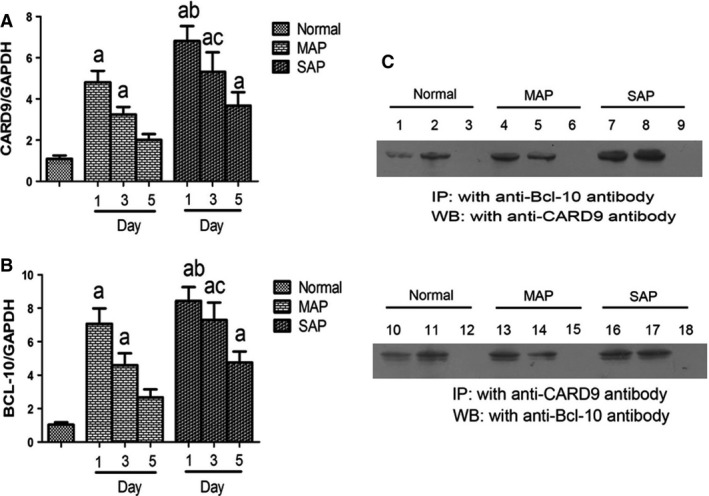
Immunoprecipitation analysis and mRNA levels in PBMCs. (**A**) Card9mRNA expression; (**B**) Bcl10 mRNA expression; (**C**) immunoprecipitation of Card9‐Bcl10 complex (input: 1, 4, 7, 10, 13, 16; anti‐Bcl‐10 immunoprecipitated complex: 2, 5, 8; anti‐Card9 immunoprecipitated complex: 11, 14, 17; lank control: 3, 6, 9, 12, 15, 18). Immunoprecipitation analysis was representative from 20 healthy volunteers, 35 MAP and 17 SAP patients. PCR results were means ± S.D. of measurement on day 1, 3 and 5 (n_controlled group_ = 20, n_MAP patients_ = 17, n_SAP patients_ = 35). a: control *versus* SAP. MAP,* P* < 0.05; b: SAP on day 1 *versus* MAP on day 1, *P* < 0.05; c: SAP on day 3 *versus* MAP on day 3, *P* < 0.05.

To explore Card9 mRNA levels, qRT‐PCR was used to determine Card9 mRNA in PBMCs. As shown in Figure 2, Card9 mRNA expression was increased on day 1, and gradually decreased in SAP patients. Compared with the control group, Card9 mRNA from day 1 in SAP and MAP patients reached 6.3 times and 4.4 times, respectively. On day 5, SAP and MAP patients still had higher Card9 mRNA than the control group.

**Figure 3:**
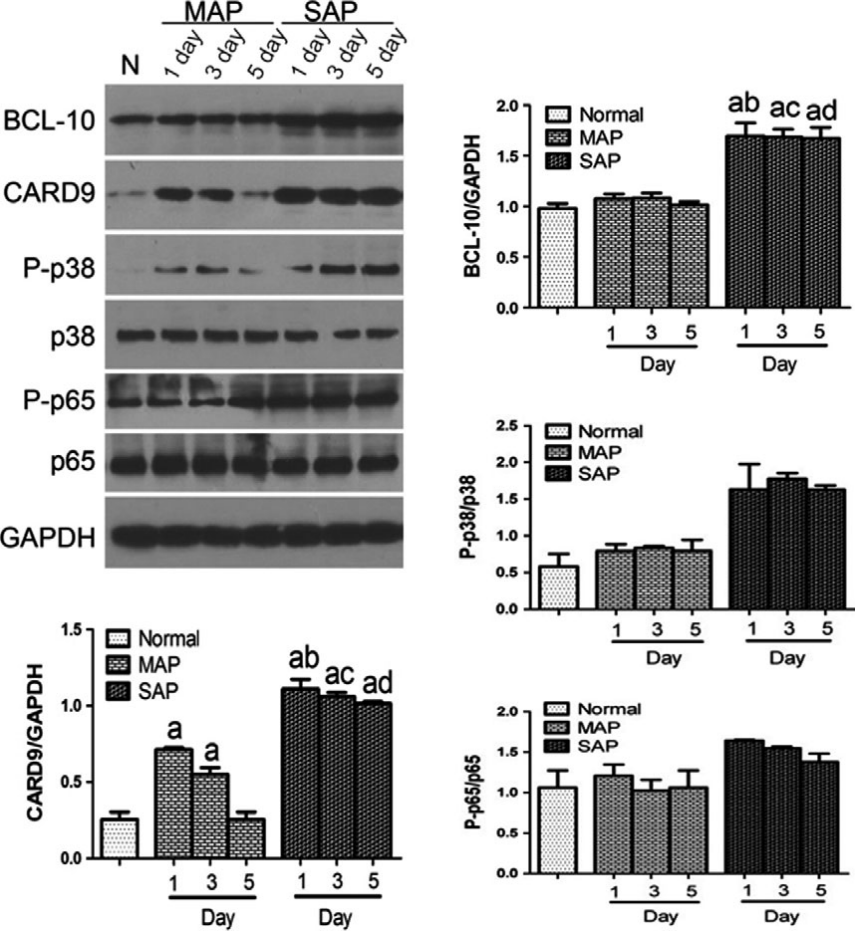
Protein expression levels in PBMCs. The protein levels of Card9, P‐p65/p65, P‐p38/p38, and GAPDH were determined by Western blot analysis (*n* = 3). a: control *versus* SAP. MAP,* P* < 0.05; b: SAP on day 1 *versus* MAP on day 1, *P* < 0.05; c: SAP on day 3 *versus* MAP on day 3, *P* < 0.05; d: SAP on day 5 *versus* MAP on day 5, *P* < 0.05.

To determine Card9 protein levels, Western blotting was applied to evaluate Card9 protein in PBMCs (Fig. 3). These results were consistent with qRT‐PCR and immunofluorescence, suggesting that Card9 levels in AP were up‐regulated at first and then gradually declined with recovery.

### Systemic cytokine responses in relation to Card9 activation in SAP patients

**Figure 4:**
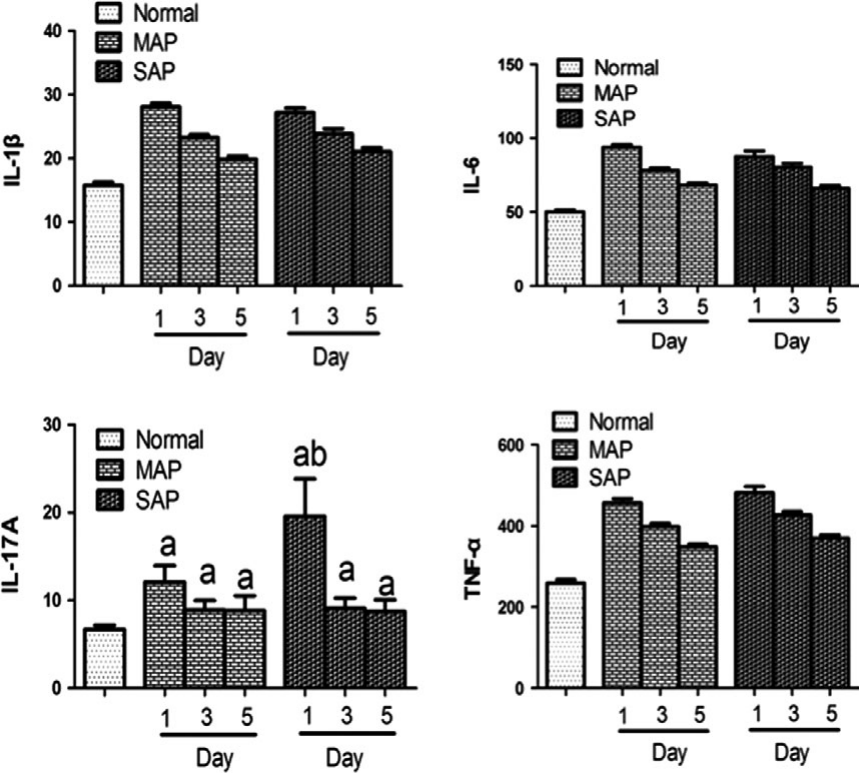
Level of cytokine in serum sample (ng/l). The protein levels of IL‐6, IL‐1β, IL‐17, and TNF‐α were determined by ELISA kits (n_controlled group_ = 20, n_MAP patients_ = 17, n_SAP patients_ = 35). a: control *versus* SAP. MAP,* P* < 0.05; b: SAP on day 1 *versus* MAP on day 1, *P* < 0.01.

Plasma levels of interleukin (IL)‐6, IL‐1β and tumour necrosis factor‐α (TNF‐α) were measured in the peripheral blood of AP patients (Fig. 4). As reported in systemic inflammation, these three cytokines were significantly elevated and peaked in SAP on day 1, then gradually declined during the course of disease recovery. There was a same trend towards the cytokine change in MAP patients (Fig. 4). Moreover, IL‐17 cytokines was present and functional in Card9 signalling pathway, which was induced and mounted by Card9 molecule activation in fungal infection 15. In this study, AP patients showed the high levels of IL‐17A on day 1 (SAP *versus* MAP, *P* < 0.05), then had a reduction in the recovery phase. This result suggested that IL‐17 cytokines were involved in aseptic inflammation responses in SAP patients.

### Effect of Card9 on the activation of NF‐κB and p38 signalling pathways in PBMCs

Considering that Card9 could significantly trigger NF‐κB and p38 MAPK activation in PBMCs 16, our study investigated whether Card9 overexpression was accompany with upstream molecule of NF‐κB and p38 activation. We first examined the expression of NF‐κB and p38 in PBMCs from SAP patients using Western blotting. Figure 3 showed that the levels of the NF‐κB p65 subunit and p38 protein in SAP groups were much higher than that of MAP and control groups (*P* < 0.05). p38 protein in MAP had a slight increase in comparison to control group (0.79 ± 0.09 *versus* 0.58 ± 0.17, *P* > 0.05) while the increase in NF‐κB p65 subunit in MAP was only found on day 1 (1.20 ± 0.14 *versus* 1.06 ± 0.21, *P* > 0.05).

As reported that the molecular mechanism of NF‐κB and p38 activation was associated in Card9‐Bcl10 complex in fungal infections 17, our study initially investigated whether the inducible formation of Card9‐Bcl10 complex was observed during aseptic inflammation phase of AP. We next examined the Card9mRNA and Bcl10mRNA in PBMCs from SAP patients using qRT‐PCR, demonstrating that their levels were markedly higher (*P* < 0.01) in SAP patients than health volunteers (Fig. 2A and B). Western blot further showed the same tendency to increase Card9 and Bcl10 protein between SAP and control groups. Figure 2C, one representative case from SAP and MAP patients, found that Card9‐Bcl10 complex significantly increased in the activated PBMCs at the early stage of SAP.

### Card9 level in PBMCs correlation with SAP

Card9 in PBMCs had a strong positive correlation with severity of SAP, suggesting that clinical improvement in SAP patients was associated with the significant decrease in Card9 levels. As shown in Figure 1, the levels of Card9 in the SAP group were significantly higher than that in the MAP and healthy volunteer groups. As the SAP patients usually alleviated the symptom by day 3, Card9 expression in PBMCs was obviously down‐regulated at 3 and 5 days after the onset of SAP.

**Figure 5:**
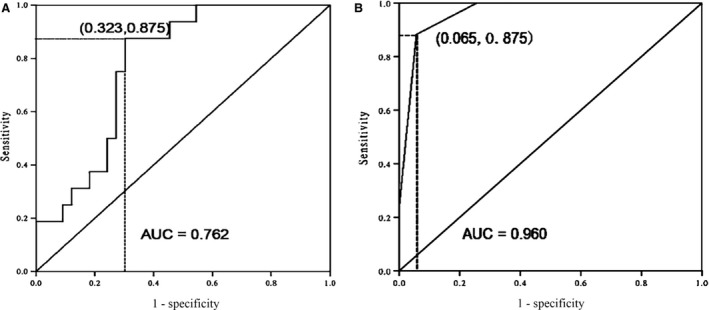
ROC curve from SAP patients. (**A**) Card9 levels (sensitivity of 87.5% and specificity of 67.7%); (**B**) Ranson score (sensitivity of 87.5% and specificity of 93.5%).

By stepwise forward logistic regression analysis, Card9 levels were identified as potential molecular marker at the early stage of SAP (*P* < 0.01). The ROC curves for Card9 and Ranson score were presented in Figure 5. Card9 levels in PBMCs for predicting SAP reached an area under curve of 0.762, high sensitivity of 87.5% and specificity of 67.7%, while Ranson score predicted SAP with area under curve of 0.960, sensitivity of 87.5% and specificity of 93.5%, respectively. These Card9 values were similar to the respective values for Ranson score, indicating Card9 had a better capable of distinguishing the SAP occurrence and lowering the disease mortality.

To validate the correlation of Card9 overexpression with SAP, clinical data from patients were further analysed. First, all patients received the same treatment on the first 3 days, because traditional Ranson classification was calculated to identify AP patients *via* using clinical data from the first 48 hrs. Especially, the first day sample did not undergo any treatment, which could completely distinguish the therapeutic bias between mild and severe cases. Second, co‐morbidities among the patients with acute pancreatitis maybe influence the body's inflammatory status and Card 9 expression in PBMCs. SAP and MAP patients in this study were presented the similar co‐morbidities, such as cholecystitis, hypertension, diabetes, cholelithiasis and hyperlipemia *etc*. Our data showed that Card9 in the SAP group were significantly higher than that in the MAP, indicating its specificity in relation to pancreatitis.

## Discussion

Emerging studies suggest that monocytes initiate inflammatory activities, which are relevant for the subsequent inflammatory cascade during SAP. These activated monocytes could recognize tissue damage to promote the recruitment of neutrophils through the production of neutrophil‐recruiting chemokines. The inhibition of monocytes activation could avoid the initiation of inflammatory responses and eliminate the subsequent inflammatory cascade reaction. As reported previously, Card9 is mainly located in monocyte‐macrophage cells, in which they are responsible for defence against bacterial and fungal infection, acting as a marked activation of NF‐κB and MAPKs signalling pathways for the inflammatory cascade reaction. In this study, we found that Card9 levels in SAP patients were obviously up‐regulated in the early period of the disease and down‐regulated in the late period. These findings showed that Card9 activation in PMBCs corretated with non‐infectious inflammation amplification at an early stage of SAP patients. It also provided the new insights into therapeutic interventions targeting monocytes activation, inducing the onset, progression and resolution of SAP.

Card9 signalling in PMBCs was involved in the recognition of bacterial, viral and fungal pathogens 18, 19. It was becoming clear that Card9 contributed to innate immune response against these infectious diseases 20. Card9^−/−^ macrophages revealed the Card9‐dependent NF‐κB and p38 control 21. Moreover, the *in vivo* production of IL‐6 and TNF‐α was reduced in pathogenic organisms infected Card9‐deficient mice, which led to impair the clearance of the intracellular pathogens and then produced a high mortality in gene‐deficient mice 22. To our knowledge, the early stage of SAP was characterized by non‐infectious inflammation. Different to the reorganization of microbial infection, PMBCs activation in SAP patients might be ignited due to an endogenous substance from the human body itself 23. Thereby, Card9 expressions in PMBCs of SAP patients maybe play a completely different role in inflammation responses compared with the previously reported infectious inflammation.

In view that Card9 was upstream molecule of NF‐κB and p38 activation, Card9 overexpression could significantly trigger NF‐κB and p38 MAPK in PBMCs from infectious inflammation. In this study, Western blotting and qRT‐PCR indicated that Card9 levels markedly increased in SAP and MAP patients in comparison with the controlled group. A marked activation of NF‐κB and p38 signalling pathways was observed in SAP patients, but not in MAP patients. Interestingly, the involvement of NF‐κB and p38 activation in Card9 up‐regulation was supported in SAP, while neither p38MAPK nor NF‐κB was activated by Card9 overexpression in MAP. Thus, this study suggested that Card9 expressions in aseptic inflammation played a different role in NF‐κB and p38 activation compared with the infectious inflammation.

Card9 main function in response to microbial antigen stimulation was to form a complex with Bcl10, which mediated Dectin1‐induced NF‐κB activation and TLR_4_ (NOD)‐induced p38 activation. However, a question remained of how to activate Card9 in PMBCs in response to aseptic inflammation in SAP. One possible explanation lied in TLR_4_ and Dectin‐1 receptors of PBMCs from SAP in the absence of infection were triggered by exogenous macromolecules. As we known, the pancreas, one of the largest digestive glands in the human body, secreted the powerful digestive enzymes to digest many kinds of substances *in vivo*. Soluble heparan sulphate, a glycosaminoglycan associated with nucleated cellular components and extracellular matrices, was released by proteases digestion in the pancreas. The rapid enzymatic degradation of heparan sulphate activated TLR_4_ in PMBCs and led to SAP aggravation, even resulted in death 24. The second possible explanation contributed to the inducible formation of Card9‐Bcl10 complex was observed in SAP patients, which may be responsible for the NF‐κB and MAPK activation in PMBCs. However, the potential function and molecular mechanism of Card9‐Bcl10 complex in SAP still need further investigation using a rat model, supporting that PMBCs in the absence of infection shared a similar complex in the production of inflammatory cytokines or not.

More evidence indicated that humans with Card9 mutation showed defects in inflammation responses, which was involved in microbial infection response and induced the cytokine release. A study on 1851 patients with inflammatory bowel disease indicated a strong association with Card9rs10870077 for the diseases, verifying Card9 was an attractive candidate gene involved in its pathogenesis and induced the production of pro‐inflammatory cytokines 25, 26. Consistent with the previous results, our data showed that an obviously increase in the production of IL‐6, IL‐1β and TNF‐α was found at an early stage of SAP patients. In addition, it had become clear that Card9 mutation was associated with the development of T‐helper cells producing IL‐17. The previous study demonstrated that Card9^−/−^ mice failed to recruit the representative cytokines IL‐17 in serum and had an increased susceptibility to candidiasis infection 27, 28. We also observed that IL‐17 elevated during the early stage of SAP, establishing that the adaptor protein Card9 coordinated IL17‐mediated immune responses in SAP pathogenesis 27.

Early prediction of SAP is needed because these patients will obtain appropriate clinical management and improve the outcome 29. Although a variety of biomarker may predict the course of SAP, it still is difficult in being useful enough to be incorporated into routine clinical use. IL‐6 has been demonstrated to have prognostic value for AP upon admission, but IL‐6 may not be perfect predictor of SAP because of the lower positive likelihood ratio with sensitivities of 56.0% and specificities of 90.6% 30, 31. Ranson's score system has been regarded as a valuable score for risk stratification and prognostic prediction in SAP patients 32. In this study, the Card9 levels in PMBCs were significantly higher in SAP patients than in MAP patients, indicative of a strong positive correlation with the severity of SAP. Regression analysis revealed that Card9 levels may be independent predictive markers with a limited number of SAP patients (*n* = 17). Compared with Ranson's score, Card9 expression had the similar predictive accuracy as seen their sensitivities and specificities. Considering that Ranson's score was a comprehensive assessment system and calculate at 48 hrs after hospital admission, Card9 levels along with hospital admission would be a simple and potential biomarker in clinical practice for predicting the severity of SAP. Therefore, it had been proposed that more SAP and MSAP patients were collected to reveal an important role of Card9 in the further study.

In conclusion, Card9 may be able to trigger NF‐κB and p38 MAPK activation in PBMCs from SAP patients, which enriched the diseases spectrum underlying Card9 signalling pathways without microbial infection.

## Conflicts of interest

The authors confirm that there are no conflicts of interest.
